# A novel strategy to identify candidate diagnostic and prognostic biomarkers for gastric cancer

**DOI:** 10.1186/s12935-021-02007-6

**Published:** 2021-07-02

**Authors:** Lei Liu, Honglin Pang, Qiao He, Biran Pan, Xiaobin Sun, Jing Shan, Liping Wu, Kaiwen Wu, Xue Yao, Yuanbiao Guo

**Affiliations:** 1 Medical Research Center, The Third People’s Hospital of Chengdu, The Affiliated Hospital of Southwest Jiaotong University, 82 Qinglong Road, Chengdu, 610031 Sichuan China; 2 College of Medicine, Southwest Jiaotong University, Chengdu, 610036 Sichuan China; 3 Department of Clinical Laboratory, Sichuan Cancer Center, School of Medicine, University of Electronic Science and Technology of China, Chengdu, 610031 Sichuan China; 4 Assisted Reproductive Center, The Maternal and Child Health Hospital of Qinzhou, Qinzhou, 535000 Sichuan China; 5 Department of Gastroenterology, The Third People’s Hospital of Chengdu, The Affiliated Hospital of Southwest Jiaotong University, Chengdu, 610031 Sichuan China

**Keywords:** Gastric cancer, Diagnosis, Prognosis, Serum biomarkers, Exosomes

## Abstract

**Background:**

Gastric cancer (GC) is one of the most common cancer worldwide. It is essential to identify non-invasive diagnostic and prognostic biomarkers of GC. The aim of the present study was to screen candidate biomarkers associated with the pathogenesis and prognosis of GC by a novel strategy.

**Methods:**

The expression level of gene higher in cancer than in adjacent non-cancer tissue was defined as “positive”, and the top 5% genes with “positive rate” were filtered out as candidate diagnostic biomarkers in three Gene Expression Omnibus (GEO) datasets. Further, a prognostic risk model was constructed by multivariate Cox regression analysis in GEO dataset and validated in The Cancer Genome Atlas (TCGA). The expression level of candidate biomarkers was determined in serum and serum-derived exosomes of GC patients. Moreover, the effect of biomarkers in exosomes on migration of GC cells was analyzed by transwell assay.

**Results:**

Ten candidate biomarkers (*AGT*, *SERPINH1*, *WNT2*, *LIPG*, *PLAU*, *COL1A1*, *MMP7*, *MXRA5*, *CXCL1* and *COL11A1*) were identified with efficient diagnostic value in GC. A prognostic gene signature consisted of *AGT*, *SERPINH1* and *MMP7* was constructed and showed a good performance in predicting overall survivals in TCGA. Consistently, serum levels of the three biomarkers also showed high sensitivity and specificity in distinguishing GC patients from controls. In addition, the expression level of the three biomarkers were associated with malignant degree and decreased after surgery in GC patients. Moreover, the expression level of AGT and MMP7 in exosomes correlated positively with serum level. The exosomes derived from serum of GC patients can promote migration of SGC‐7901 cells. After neutralized the expression level of three proteins in exosomes with antibodies, the migration of GC cells was obviously suppressed.

**Conclusions:**

Our findings provided a novel strategy to identify diagnostic biomarkers based on public datasets, and suggested that the three-gene signature was a candidate diagnostic and prognostic biomarker for patients with GC.

## Background

Gastric cancer (GC) is one of the most common cancer worldwide and is responsible for over 1,000,000 new cases in 2018 and an estimated 783,000 deaths, making it the fifth most frequently diagnosed cancer and the third leading cause of cancer death. The incidence rates of GC are markedly elevated in Eastern Asia [1]. The 5-year survival of GC is low because more than 80% of patients are diagnosed at an advanced stage and lose the opportunity for the most effective surgical treatment [2, 3]. Thus, screening and early diagnosis is the most effective way to improve the survival rate.

The diagnosis of gastric cancer relies on endoscopy and biopsy, but the invasiveness of these means leads to poor compliance and potential risks to patients. Blood markers play an important role in disease screening and diagnosis due to its economic, convenient and minimally invasive characteristics. In gastric cancer, several blood markers have been used for diagnosis, determination of the clinical stage, evaluation of treatment responses and screening for recurrence after successful therapy [4]. Although many biomarkers for GC including carbohydrate antigen 72–4 (CA72-4), alphafetoprotein, carbohydrate antigen 12–5 (CA12-5), SLE, BCA225, hCG and pepsinogenI/II have been reported, carcinoembryonic antigen (CEA) and carbohydrate antigen 19–9 (CA19-9) are still the most frequently used biomarkers in clinical practice for GC [5]. However, low rates of sensitivity and specificity prevent the use of any of these serum markers in diagnosis of GC [4]. As current non-invasive tests are insufficient for GC screening or diagnosis, the discovery of alternative biomarkers is necessary, especially blood biomarkers.

In recent years, microarray and high throughput sequencing technologies have been used for discovering diagnostic or prognostic biomarkers of cancers [6]. Usually, based on the difference of genes expressed in tumor tissue and normal controls, genes with the most difference fold change (FC) are considered for candidate biomarkers. However, the most critical feature of a diagnostic marker is that it presents universally in almost all the cancer patients but not at the healthy individuals, although the marker is probably not the most different molecule between the tumor and healthy. Therefore, we think the positive ratio of a tumor biomarker, which is expressed higher in cancer than in adjacent non-cancer tissue, presents in the cancer patients determines its sensitivity. Based on this thought, a novel strategy was established for mining candidate blood biomarkers from the GEO datasets.

In the present study, three GEO datasets with large GC cohorts, which contain paired cancer and adjacent non-cancer tissues, were employed for candidate biomarkers screening using the mentioned mining method above. Then, we performed gene ontology (GO) term and find 16 proteins were located in extracellular region. The diagnostic value of the genes was validated using receiver operating characteristic (ROC) curve in TCGA and GEO datasets, and 10 genes were picked out for further analyses. Based on the Cox regression analysis, three candidate genes (*AGT*, *SERPINH1* and *MMP7*) were identified which associated with the overall survival (OS) of GC patients. The expression level of three candidate biomarkers in serum were associated with tumor malignant degree and decreased after surgery in GC patients. In additional, the expression level of three candidate biomarkers in exosomes correlated positively with serum level. Moreover, the three proteins probably promote progress of GC through exosomes. This study identified novel blood biomarkers in diagnosis and prognosis of GC.

## Methods

### Datasets and preprocessing

The gene expression data used in this study were obtained from GEO and TCGA database. Three GEO datasets (GSE66229, GSE27342, GSE63089) were selected to screen the candidate genes for diagnosis of GC, which consists of paired tumor tissue and adjacent non-cancer tissue with large sample size. The datasets TCGA and GSE54129 were employed to validate the efficiency of the candidate genes in diagnosis of GC. GSE15459 was selected to construct the prognosis risk model of GC and the risk model was tested in TCGA database. The Raw CEL files of the 5 GEO datasets were downloaded and the Robust Multichip Average (RMA) algorithm was used for background adjustment, quantile normalization and log-transformation. In addition, the gene expression profiles of TCGA database was transformed to the base-2 logarithm for further analysis.

### Receiver operating characteristic (ROC) curve analysis

The datasets TCGA and GSE54129 were used for validating the efficiency of the candidate genes in diagnosis of GC. ROC curve analysis was performed to evaluate the efficiency of the candidate genes in diagnosis of GC. The area under the curve (AUC) value was calculated and used for evaluating the diagnostic value of these genes for GC diagnosis. The candidate genes with AUC value greater than 0.6 in the both datasets were regarded as acceptable for diagnosis and selected for further study [7, 8].

### Construction of prognostic risk model

GSE15459 was used as the training dataset to construct the prognosis risk model. The patients without overall survival (OS) data were excluded, and finally 190 GC patients were included for analysis. First, the univariate Cox regression analysis was used for determining the association between the expression level of the ten candidate genes and overall survival (OS) of GC patients, and the genes with *P*-value < 0.05 were selected for multivariate Cox regression analysis to identify prognostic genes. The coefficients of each prognostic gene were performed to construct the prognostic risk model. The formula is as follows: risk score = coefficient of gene_1_ × expression of gene_1_ + coefficient of gene_2_ × expression of gene_2_ + … coefficient of gene_n_ × expression of gene_n_.

### Survival analysis

The risk model was used for validating prognosis of GC patients in datasets GSE15459 and TCGA. The risk score was calculated in each patient of GC and the patients were classified into low-risk and high-risk groups based on the median risk score. The Kaplan‐Meier survival analysis was performed for the patients in different groups. Furthermore, the time‐dependent ROC curve was performed by R package “survival ROC” to assess the predictive accuracy of the prognostic risk model. The AUC was calculated to measure the predictive ability of the candidate genes for time-dependent cancer death.

### Patients

To validate the diagnosis and prognosis value of three genes in GC, the blood samples from GC patients and healthy volunteers were collected at the Sichuan Cancer Hospital between October 2018 and July 2020. The GC patients were newly diagnosed without received any chemotherapy or radiation therapy and histologically confirmed by two different pathologists. The subjects in healthy controls group showed no cancer and other serious disease in the physical examination. The postoperative blood samples were collected from 25 patients around 1 month after surgery. The present study was approved by the Ethics Committee of Sichuan Cancer Hospital. Informed consent was obtained from all of the participants before the start of the study.

### Blood sample collection and detection

Venous blood was collected in the morning before breakfast from all of the subjects. Blood samples were allowed to clot at room temperature for 30 min, and then centrifuged at 2000*g* for 10 min at 4 °C to separate serum. Then the serum was aliquoted and stored at – 80 °C for further study. The concentration of candidate markers AGT, SERPINH1 and MMP7 in serum were determined by a quantitative sandwich enzyme-linked immunosorbent assay (ELISA) kit (USCN, Wuhan, China) according to the manufacturer’s instructions.

### Exosomes isolation from serum

The exosomes were isolated from serum by precipitation or size exclusion chromatography (SEC). For precipitation, ExoQuick precipitation was carried out according to manufacturer’s instructions (System Biosciences). Briefly, one milliliter of serum was thawed and centrifuged at 1500*g* for 10 min. The supernatant was collected and recentrifuged at 10,000*g* for 30 min. The supernatant was then incubated with ExoQuick for 30 min at 4 °C. The ExoQuick/serum sample was then centrifuged at 1500*g* for 30 and the pellet was resuspended in 200 µL of PBS. For SEC, 500 µL of serum was thawed and recentrifuged at 10,000*g* for 30 min. The clarified serum was overlaid on qEV size exclusion columns (Izon, New Zealand) followed by elution with PBS. The eluate was collected in 13 sequential fractions of 1 mL. Each fraction was aliquoted and stored at − 80 °C for subsequently study.

### Identification of exosomes

The concentration and size distribution of particles in collected fractions was measured by Nanoparticle tracking analysis with ZetaView (Particle Metrix, Meerbusch, Germany). Each sample was diluted 1:1000 to 1:10,000 in PBS to get the ideal measurement concentration (50–200 particles/frame). The instrument set to a specific analysis parameter: Minimum brightness: 20, Maximum area: 1000, Minimum Area: 5, Laser Wavelength: 488 nm. Each measurement scan at 11 different positions, and after capture, the videos were analyzed by the software ZetaView (version 8.05.11).

Exosomes were visualized using transmission electron microscopy (TEM) (JEM-1400, JEOL, Japan). Briefly, freshly isolated exosomes were put on a copper grid and keep at room temperature for 5 min, then the grids were stained with 2% (v/v) uranyl acetate and the exosome samples were examined immediately.

Isolated exosomes were detected for the presence of exosomal markers TSG101 and CD63 by Western blotting. The Protein concentration was detected by bicinchoninic acid assay (BCA assay) (ThermoScientific, IL, USA). The equal amounts (30ug) of precipitated exosomes or equal volume (20 ul) of SEC fractions were loaded on 12% SDS-PAGE gels. Proteins were transferred to PVDF membrane (Millipore, MA, USA) and the membranes were incubated with primary antibodies TSG101 and CD63 (Proteintech, Wuhan, China) at 4 °C overnight. After incubated with horseradish peroxidase-conjugated species-specific secondary antibodies, the membranes were visualized with enhanced chemiluminescence reagent (Millipore, MA, USA).

### Cell culture and uptake of exosomes

The GC cell line SGC‐7901 were purchased from the cell bank of the Chinese Academy of Sciences (Shanghai, China) and cultured in RPMI-1640 medium supplemented with 10% FBS, 100 U/ml penicillin and 100 mg/ml streptomycin. Cells were cultured at 37 °C under a humidified atmosphere including 5% CO_2_. For tracking exosomes, purified exosomes were incubated with 1uM fluorescent dye DiI (AAT Bioquest, CA, USA) according to the manufacturer's guidelines. After washed with PBS, the DiI-labeled exosomes were co-cultured with SGC7901 for 24 h. The uptake of exosomes was observed by laser confocal microscope (SpinSR10, OLYMPUS, Japan).

### Cell migration assay

Cell migration assay was performed using a 24-well migration chamber (Corning, NY, USA) with or without exosomes. In the exosomes group, exosomes derived from serum of GC patients were treated with neutralizing antibodies (anti-AGT, anti- SERPINH1, anti-MMP7 respectively or combined) or isotype control for 30 min, and then washed with PBS. The pretreated exosomes (30ug) and 5 × 10^4^ SGC‐7901 cells were co-cultured in the top chamber. The bottom chamber was filled with 600 μl medium containing 20% exosomes-free FBS. After incubation for 48 h, the cells remaining at the upper surface of the membrane were removed with a cotton swab, and those that adhered to the lower surface were fixed with paraformaldehyde and stained with crystal violet. Five fields were selected randomly to count the cells invaded through the membrane and imaged under a microscope (Leica Microsystems, Wetzlar, Germany) with magnification of 100 times. Each experiment was performed three times independently and the mean number of invaded cells were used for Transwell assay assessment.

### Statistical analysis

The univariate and multivariate Cox regression analysis was used for determining predictive factors for GC prognosis. The Mann–Whitney U test was used for analyzing the difference of concentration of candidate markers between control and GC groups. The ROC curve was used for evaluating the diagnosis value of candidate markers. The Pearson’s chi-square test was used for analyzing the associations between concentration of candidate markers and clinicopathological characteristics. The paired Student’s t-tests was used for comparison of concentration of candidate markers between pre- and postoperative samples in GC. Pearson correlation assay was used for analyzing the expression level of three genes between serum and exosomes. All analysis was performed using SPSS 20.0 software (IBM Corporation, NY, USA) or GraphPad Prism 7.0 (GraphPad Software Inc., CA, USA). *P* < 0.05 was considered as significant difference.

## Results

### Screening the candidate genes from GEO datasets of gastric cancers

**Fig. 1:**
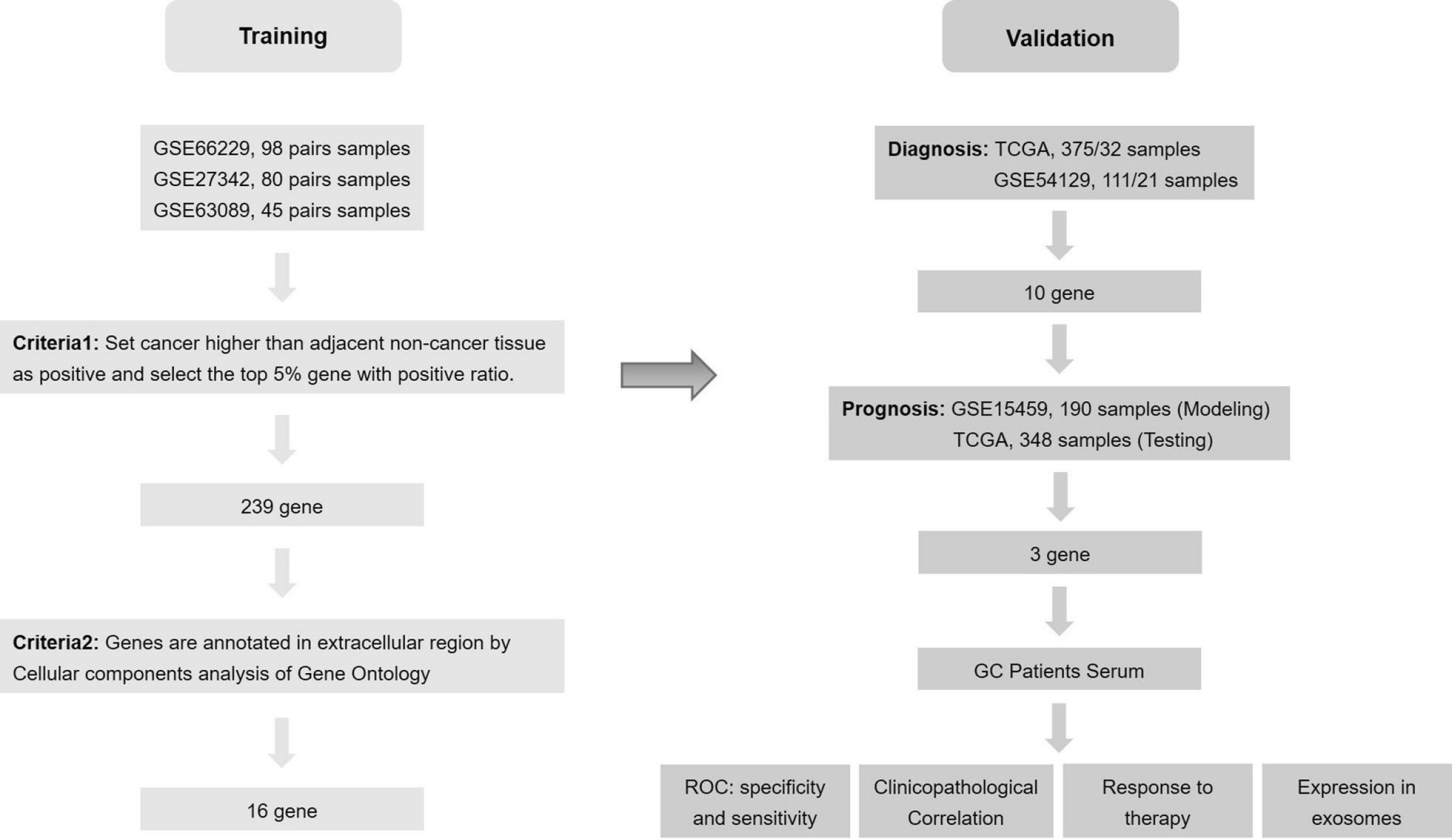
Flowchart for the novel strategy to identify candidate diagnostic and prognostic biomarkers in GC. TCGA, The Cancer Genome Atlas; ROC, receiver operating characteristic

The most critical feature of a diagnostic blood marker is that it presents universally in almost all cancer patients but not at all or at lower levels in healthy individuals. Therefore, the expression level of gene higher in cancer than in adjacent non-cancer tissue were define as positive, and we believe that the positive rate of candidate biomarkers in cancer patients is as important as its difference in concentration between cancer patients and healthy people. A series of research were conducted following the schematic diagram in Fig. 1 in the hope of finding new blood candidate biomarkers.

**Fig. 2:**
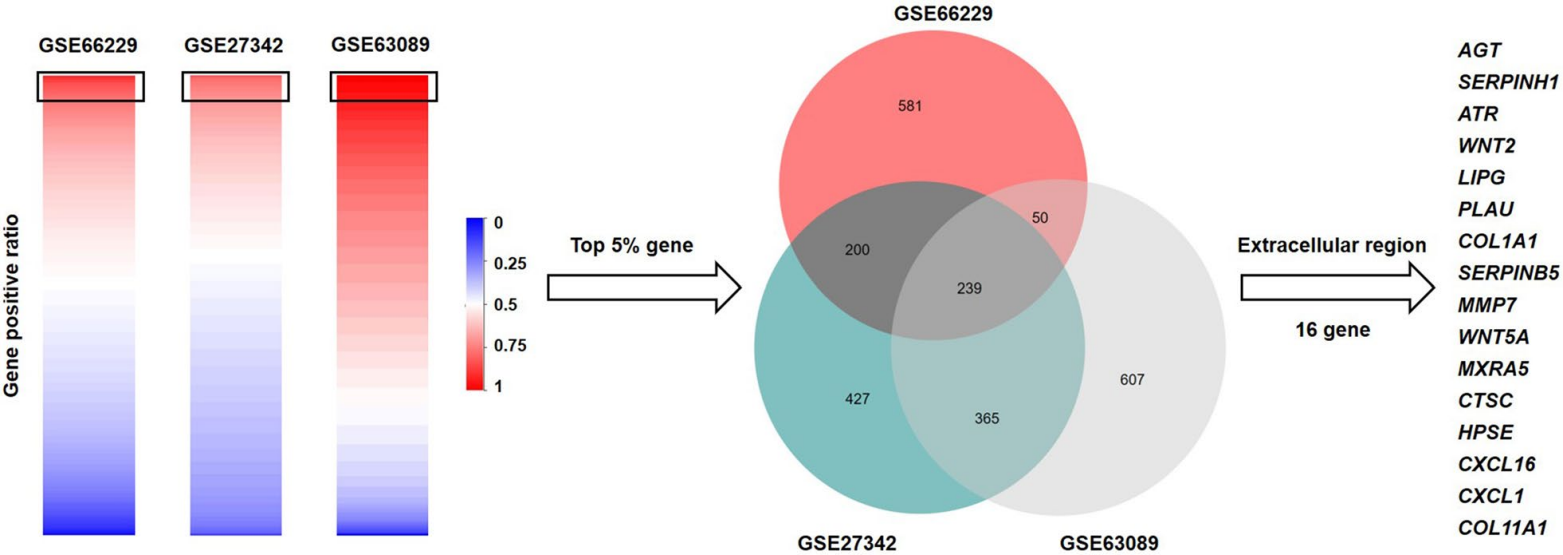
Venn diagram of the top 5% of positive rates genes among the three training GEO datasets

Three GEO datasets with large sample sizes, GSE66229 (n = 98), GSE27342 (n = 80) and GSE63089 (n = 80), were employed for candidate biomarkers screening. After ranking genes by their positive rates in cancer cases, the top 5% genes of each dataset were filtered out. GSE66229, GSE27342 and GSE63089, respectively, yielded 1070, 1231 and 1261 genes that were universally expressed in cancer tissue, and 239 overlapping genes were identified. To uncover the blood biomarkers, we focused on the extracellular proteins. The 239 genes were annotated by Cellular Components analysis of Gene Ontology (GO), and 16 proteins were located in extracellular region: *AGT, SERPINH1, ATR, WNT2, LIPG, PLAU, COL1A1, SERPINB5, MMP7, WNT5A, MXRA5, CTSC, HPSE, CXCL16, CXCL1* and *COL11A1* (Fig. 2).

### Diagnostic evaluation of 16 candidate genes

To validate the efficiency of the novel strategy for biomarkers screening, the efficiency of 16 candidate genes in distinguish GC from NC tissues was analyzed in TCGA (375 GC tissues and 32 normal tissues). The results showed that all of the 16 candidate genes performed excellent. To analyze the diagnostic efficiency of these genes in GC, the ROC curve analysis was performed with dataset GSE54129 (111 GC tissues and 21 normal tissues). There are ten candidate genes with AUC value greater than 0.6 in GSE54129 dataset: *AGT, SERPINH1, WNT2, LIPG, PLAU, COL1A1, MMP7, MXRA5, CXCL1* and *COL11A1* (Table 1). These ten genes were used for further constructing of cox regression model.

**Table 1:** Area under the curve of candidate biomarkers in distinguishing controls from GC patient**s**

Gene	TCGA					GSE54129				
	AUC	Std. error	P-value	95% CI		AUC	Std. error	P-value	95% CI	
				Lower	Upper				Lower	Upper
AGT	0.717	0.040	< 0.001	0.639	0.795	0.617	0.049	0.089	0.522	0.713
SERPINH1	0.913	0.020	< 0.001	0.873	0.952	0.957	0.016	< 0.001	0.925	0.989
ATR	0.908	0.026	< 0.001	0.856	0.959	0.328	0.049	0.013	0.232	0.425
WNT2	0.946	0.014	< 0.001	0.919	0.973	0.991	0.007	< 0.001	0.978	1.000
LIPG	0.829	0.032	< 0.001	0.766	0.892	0.890	0.039	< 0.001	0.813	0.966
PLAU	0.892	0.018	< 0.001	0.858	0.927	0.913	0.025	< 0.001	0.864	0.962
COL1A1	0.930	0.015	< 0.001	0.901	0.959	0.972	0.014	< 0.001	0.945	0.999
SERPINB5	0.661	0.059	0.003	0.545	0.776	0.403	0.057	0.159	0.292	0.514
MMP7	0.787	0.038	< 0.001	0.713	0.861	0.733	0.047	0.001	0.641	0.826
WNT5A	0.719	0.057	< 0.001	0.607	0.831	0.550	0.051	0.465	0.451	0.650
MXRA5	0.823	0.025	< 0.001	0.775	0.871	0.782	0.040	< 0.001	0.703	0.860
CTSC	0.706	0.050	< 0.001	0.609	0.804	0.545	0.058	0.512	0.432	0.658
HPSE	0.750	0.053	< 0.001	0.646	0.853	0.469	0.060	0.652	0.351	0.587
CXCL16	0.796	0.037	< 0.001	0.723	0.870	0.462	0.054	0.582	0.356	0.568
CXCL1	0.830	0.035	< 0.001	0.762	0.898	0.783	0.046	< 0.001	0.693	0.874
COL11A1	0.924	0.016	< 0.001	0.893	0.955	0.911	0.025	< 0.001	0.862	0.959

*AUC* area under curve, *Std.* standard, *CI* confidence interval

### Assess the prognostic risk of the biomarker candidates in the training dataset

To explore whether the 10 genes have the potential to predict prognosis, the correlation between gene expression level and survival time were analyzed by univariate and multivariate Cox proportional hazards regression in training dataset GSE15459 (n = 190). A total of eight genes significantly correlated with survival time (*P* < 0.05) were identified by the univariate Cox proportional hazards regression model. Among these genes, *AGT*, *SERPINH1* and *MMP7* displayed significant prognostic values by the multivariate Cox proportional hazards regression model (Table 2). The three genes then were used as biomarker panel for the diagnostic and prognostic indication of gastric cancers.

**Table 2:** Association between the ten gene signature and overall survival of GC patients in GSE15459

Gene	Univariate analysis		Multivariate analysis		
	HR (95% CI)	P-value	HR (95% CI)	P-value	Coefficient
AGT	1.269 (1.092–1.476)	0.002	1.197 (1.036–1.384)	0.015	0.180
SERPINH1	1.678 (1.289–2.184)	< 0.001	1.722 (1.296–2.289)	< 0.001	0.544
WNT2	1.185 (1.006–1.395)	0.042			
LIPG	1.176 (0.973–1.421)	0.094			
PLAU	1.406 (1.163–1.699)	< 0.001			
COL1A1	1.481 (1.187–1.849)	0.001			
MMP7	1.164 (1.061–1.278)	0.001	1.144 (1.044–1.254)	0.004	0.135
MXRA5	1.245 (1.026–1.511)	0.027			
CXCL1	0.958 (0.853–1.075)	0.464			
COL11A1	1.188 (1.068–1.322)	0.002			

*HR* hazard ratio, *CI* confidence interval

### Testing the prognostic risk models of the three-gene panel in the training and validating datasets

**Fig. 3:**
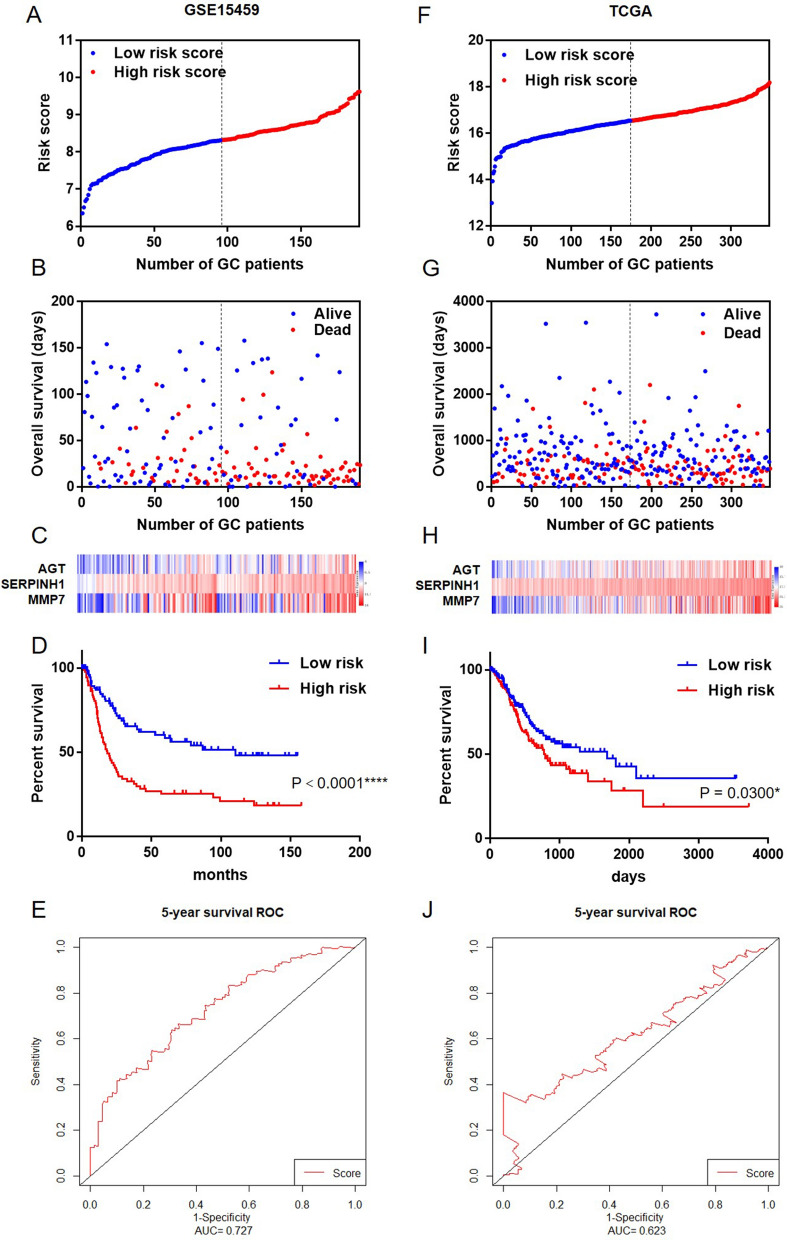
Kaplan–Meier estimates of the survival for patients using the three-gene signature. **A**, **F** Three-gene signature risk score distribution in the training and validation datasets. The black dotted line represents the median three-gene signature risk score cutoff dividing patients into low-risk and high-risk groups. **B**, **G** GC patients’ survival status in the training and validation datasets. **C**, **H** Heatmap of the three gene expression profiles in the training and validation datasets. Rows represent genes, and columns represent patients. Red: high expression; Blue: low expression. **D**, **I** Kaplan–Meier analysis for the overall survival of GC patients in the training and validation datasets. **E**, **J** The ROC curves for predicting OS in GC patients by the three-gene signature risk score

To investigate whether the three-gene panel could provide an accurate prediction of OS in GC patients, the prognostic risk scores of the panel was formulated in the training dataset (GSE15459) and validating dataset (TCGA) based on the coefficient of the three genes in multivariate analysis: Risk score =  (0.180 × expression value of *AGT*) +  (0.544 × expression value of *SERPINH1*) +  (0.135 × expression value of *MMP7*). In the training dataset, a total of 190 patients were divided into a high-risk group (*N* = 95) and a low-risk group (*N* = 95) according to the median risk score (Fig. 3A). The GC patients with high-risk scores had lower OS rates than those with low-risk scores (Fig. 3B). Moreover, tumor tissues obtained from patients with high-risk scores tended to express high level of prognostic genes (*AGT*, *SERPINH1* and *MMP7*) (Fig. 3C). As expected, GC patients with high-risk scores had lower OS than those with low-risk scores (*P* < 0.0001) (Fig. 3D). The prognostic gene signature presented a good performance in survival prediction, as the AUC was 0.727 for 5-year OS (Fig. 3E).

To confirm our findings, the prognostic power of the three-gene panel was further evaluated in the validating dataset. According to the same risk formula and using the median risk score as the cutoff point, patients in TCGA dataset were divided into high-risk group (n = 174) and low-risk group (n = 174). Consistent with the findings in training dataset, patients in the high-risk group suffered significantly poorer OS than those in the low-risk group (*P* < 0.05). The predictive power of prognosis was consistent with those observed in the training dataset (Fig. 3F–J). These results indicated that the three-gene panel showed a good performance in prognosis assessment.

### Prognostic risk model of the three-gene panel is independently associated with OS of GC patients in datasets

Next, we evaluated whether the three-gene panel was an independent predictor of GC patient’s survival. The univariate and multivariate Cox regression analysis were performed in GSE15459 dataset, and the univariate Cox regression analysis showed that the prognostic risk score and the pathological stage significantly correlated with OS of GC patients (*P* < 0.05). Furthermore, multivariate Cox regression analysis was performed using the pathological stage and the risk scores. The results showed that the prognostic risk score and the pathological stage independently correlated with OS of GC patients (*P* < 0.05). In TCGA dataset, the univariate Cox regression analysis showed that the three-gene panel risk score, tumor invasion, lymph node invasion, metastasis and the pathological stage significantly correlated with OS of GC patients (*P* < 0.05). Furthermore, the multivariate Cox regression analysis showed that the tumor invasion and metastasis significantly correlated with OS (*P* < 0.05), and there is a similar tendency in the three-gene panel risk score (HR = 1.296, 95% CI: 0.918–1.829, P = 0.141) (Table 3). These results demonstrate that the three-gene panel prognostic risk model could be independently used for predicting OS in GC patients.

**Table 3:** Univariate and multivariate Cox regression analyses in GSE15459 and TCGA dataset

Variables	Univariate analyses		Multivariate analyses	
	HR (95% CI)	P-value	HR (95% CI)	P-value
GSE15459				
Three gene risk score	2.470 (1.620–3.767)	< 0.001	1.806 (1.178–2.771)	0.007
Gender	0.713 (0.462–1.101)	0.127		
Age	0.966 (0.645–1.445)	0.865		
Stage	6.520 (3.597–11.816)	< 0.001	5.684 (3.110–10.389)	< 0.001
TCGA				
Three gene risk score	1.446 (1.034–2.022)	0.031	1.296 (0.918–1.829)	0.141
Gender	0.917 (0.644–1.306)	0.630		
Age	0.933 (0.669–1.301)	0.682		
Tumor invasion	1.880 (1.218–2.902)	0.004	2.218 (1.405–3.500)	0.001
Lymph node invasion	1.727 (1.145–2.605)	0.009	1.482 (0.884–2.485)	0.135
Metastasis	1.931 (1.067–3.496)	0.030	1.893 (1.021–3.508)	0.043
Pathological stage	1.518 (1.082–2.128)	0.016	1.196 (0.768–1.861)	0.428
Histological grade	1.313 (0.924–1.866)	0.129		

*HR* hazard ratio, *CI* confidence interval

### Performance of circulating levels of the three proteins in the diagnosis of GC patients

**Fig. 4:**
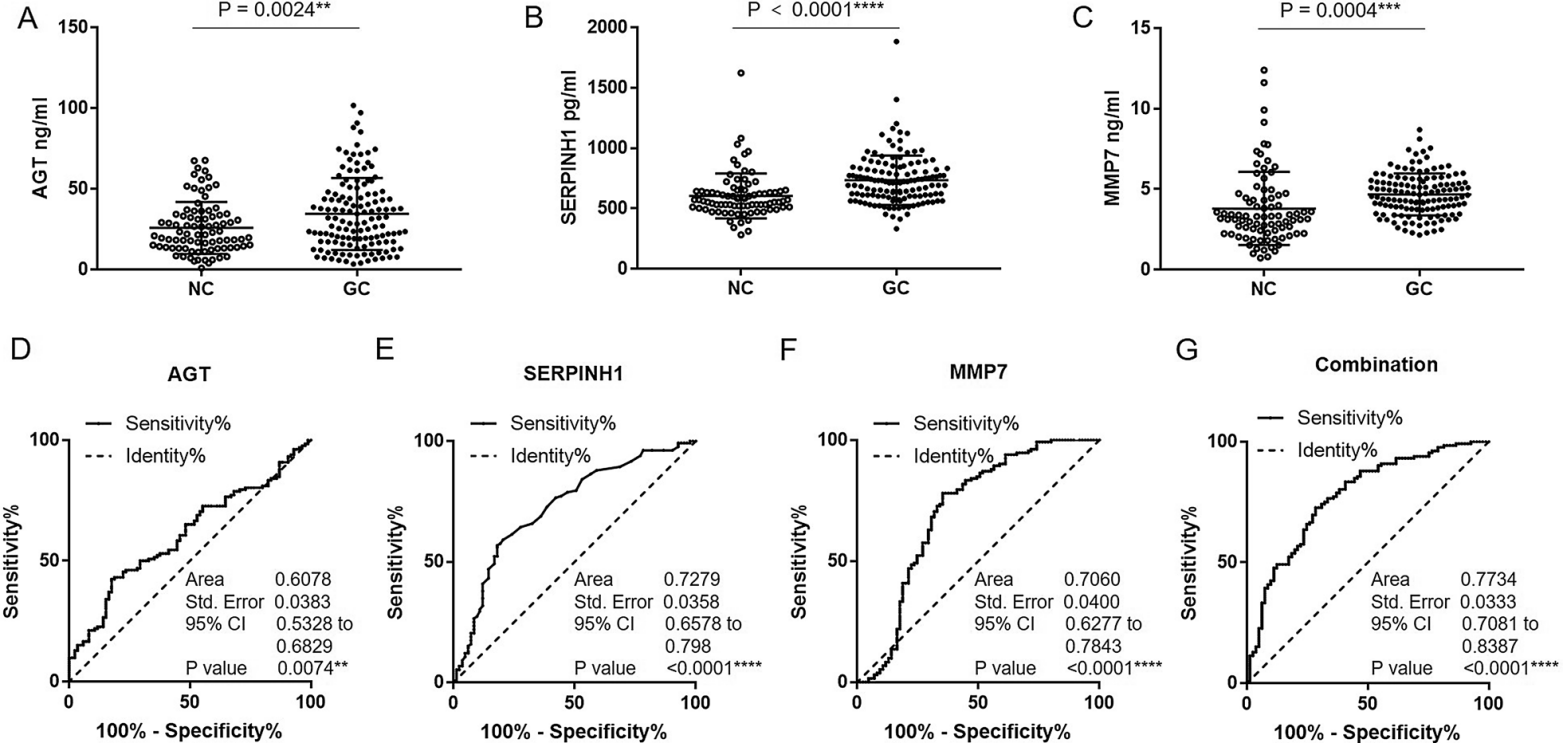
The potential diagnostic value of serum level of three candidate biomarkers in GC patients. (A-C) The expression levels of three candidate biomarkers in serum from NC and GC patients. (D-G) The potential diagnostic value of serum level of three candidate biomarkers in GC patients by ROC curve analysis. ***P < 0.001, ****P < 0.0001

In order to assess the diagnostic capability of the three proteins, the circulating levels of them were quantified in 132 GC patients and 86 controls, whose age and sex were matched. According to the grade and WHO classification, there were 99 cases of well or moderately differentiated subtype; 24 cases of poorly differentiated subtype and 9 cases of signet ring cell subtype. The ELISA results showed that the concentration of AGT, SERPINH1 and MMP7 in GC patients (34.51 ± 22.35 ng/mL, 733.90 ± 204.65 pg/mL and 4.67 ± 1.30 ng/mL, respectively) are significantly higher than those in the control group (25.90 ± 16.08 ng/mL, 604.41 ± 185.04 pg/mL and 3.80 ± 2.27 ng/mL, respectively) (Fig. 4A–C). The diagnostic performance of the three proteins was evaluated by ROC curve. The AUCs of ROC of AGT, SERPINH1 and MMP7 are 0.6078, 0.7279 and 0.706 for distinguishing GC patients from controls, respectively. The optimal diagnostic cutoff value of AGT, SERPINH1 and MMP7 are 37.10 ng/mL, 677.00 pg/mL and 3.66 ng/mL respectively, at which with sensitivity (42.42%, 56.82% and 78.03%) and specificity (82.35%, 81.93% and 64.71%). Further, the three-protein panel showed more effective performance with sensitivity (72.73%) and specificity (71.60%) (Fig. 4D–G). These results indicate that the three-protein panel in serum displayed a high potential in the diagnosis of GC.

### Correlations between clinicopathological characteristics and circulating levels of the three proteins in GC patients

Then, serum protein levels of the three genes were detected to study their clinicopathological values. GC patients were divided into 2 groups with high or low concentration of AGT, SERPINH1 and MMP7 by their median values, individually. High-AGT patients showed larger tumor size (χ^2^ = 9.008, *P* = 0.003), more depth of tumor invasion (χ^2^ = 4.281, *P* = 0.039) and more advanced TNM stage (χ^2^ = 5.961, *P* = 0.0015) than low-AGT patients. High-SERPINH1 patients also showed more depth of tumor invasion (χ^2^ = 5.979, *P* = 0.014) and more advanced TNM stage (χ^2^ = 9.854, *P* = 0.002) than the low-SERPINH1 ones. Consistently, compared to the low-MMP7 patients, patients with high MMP7 showed larger tumor size (χ^2^ = 7.013, *P* = 0.008), more depth of tumor invasion (χ^2^ = 5.979, *P* = 0.014) and more advanced TNM stage (χ^2^ = 5.961, *P* = 0.015). When evaluating the prognosis of GC patients by combination of the three proteins in serum, it still showed good performance (Table 4). These data suggested that the circulating levels of three proteins could be a potential prognostic.

**Table 4:** Correlation between serum level of the three biomarkers and clinicopathological characteristics in GC patients

Characteristics	AGT level			SERPINH1 level			MMP7 level			Score 1 level			Score 2 level		
	Low (n = 66)	High (n = 66)	P-value	Low (n = 66)	High (n = 66)	P-value	Low (n = 66)	High (n = 66)	P-value	Low (n = 66)	High (n = 66)	P-value	Low (n = 66)	High (n = 66)	P-value
Gender															
Male	45	45	1.00	49	41	0.135	47	43	0.455	49	41	0.135	51	38	0.196
Female	21	21		17	25		19	23		17	25		19	23	
Age (years)															
≤ 65	37	42	0.375	41	38	0.594	40	39	0.859	41	38	0.594	42	36	0.909
> 65	29	24		25	28		26	27		25	28		28	25	
Tumor size (cm)															
≤ 4	47	30	0.003	42	35	0.217	46	31	0.008	42	35	0.217	50	27	0.002
> 4	19	36		24	31		20	35		24	31		20	34	
Tumor invasion															
T1–T2	26	15	0.039	27	14	0.014	27	14	0.014	27	14	0.014	30	11	0.002
T3–T4	40	51		39	52		39	52		39	52		40	50	
Lymph node invasion															
Negative	27	22	0.368	27	22	0.368	27	22	0.368	27	22	0.368	30	19	0.167
Positive	39	44		39	44		39	44		39	44		40	42	
Metastasis															
Negative	57	54	0.475	58	53	0.234	59	52	0.096	58	53	0.234	63	47	0.044
Positive	9	12		8	13		7	14		8	13		7	14	
TNM stage															
I–II	38	24	0.015	40	22	0.002	38	24	0.015	40	22	0.002	42	20	0.002
III–IV	28	42		26	44		28	42		26	44		28	41	

Score 1 =  (0.180 × concentration of AGT) +  (0.544 × concentration of SERPINH1) +  (0.135 × concentration of MMP7), patients were divided into a high-risk group and a low-risk group according to the median of score 1

Score 2, two or more of the three proteins are high-expressed, the patients were divided into a high-risk group; otherwise, the patients were divided into a low-risk group

### Circulating levels of three proteins were significantly decreased in postoperative GC patients

**Fig. 5:**
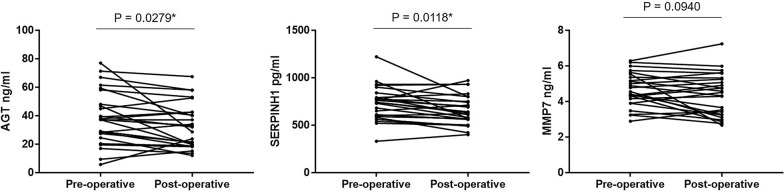
Changes of serum level of three candidate biomarkers in GC patients undergoing surgery. *P < 0.05

For monitoring the efficiency of treatment in GC patients, the changes of circulating levels of three proteins between paired pre- and post-operative serum specimens were analyzed in 25 patients with GC. The serum level of AGT and SERPINH1 were significantly decreased one month after radical operation (*P* < 0.05). There is a similar tendency in the serum MMP7, however, which with no statistical significance (Fig. 5).

### The expression level of three proteins in serum-derived exosomes from GC patients

**Fig. 6:**
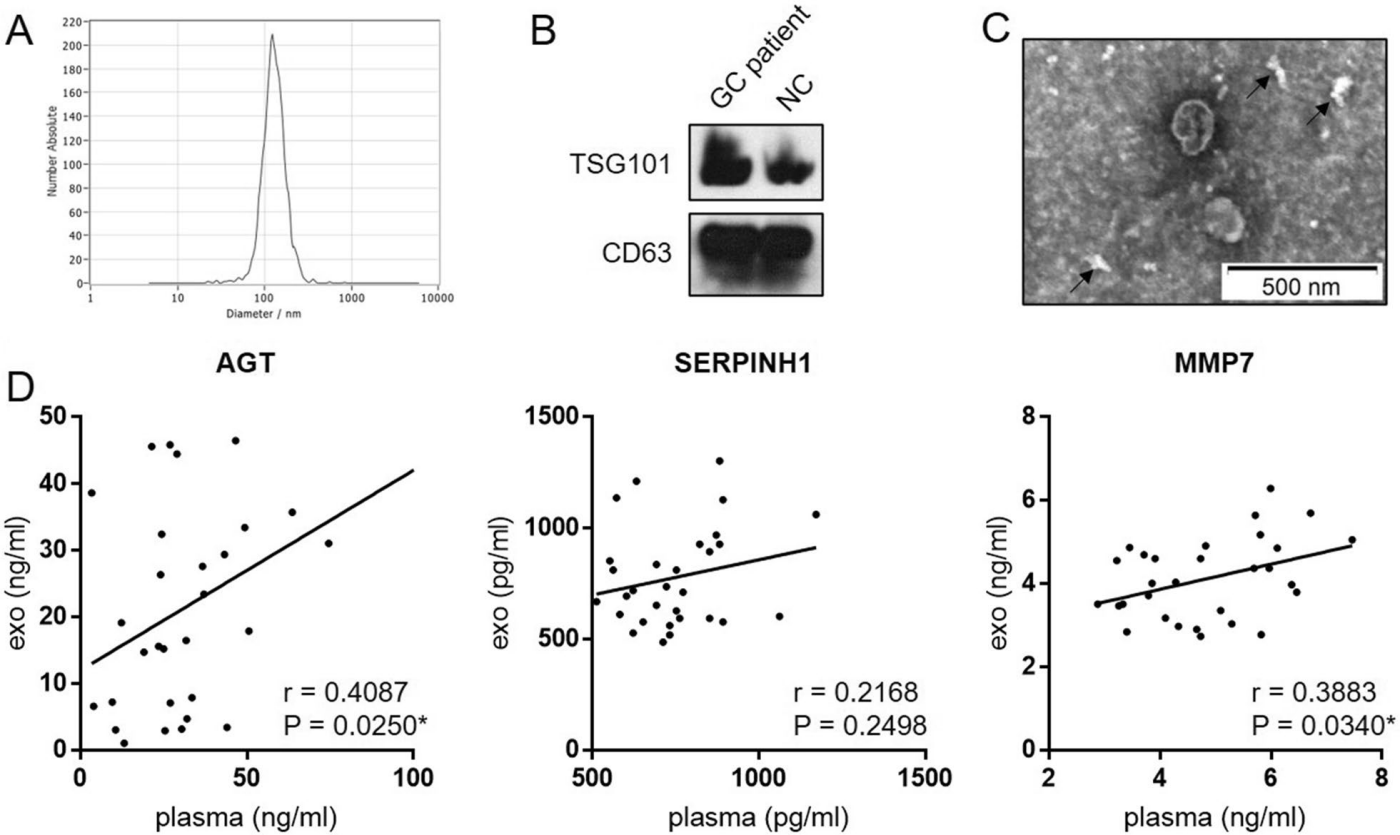
The correlation of the expression levels of three candidate biomarkers between serum and exosomes. **A**–**C** The exosomes which isolated by ExoQuick precipitation were identified by NTA, Western blotting and TEM. The black arrows indicate impurities. **D** The expression levels of three candidate biomarkers between serum and exosomes

Given the three genes located in extracellular region by GO annotation, we wanted to know their protein levels in serum exosomes. The exosomes derived from serum were isolated by ExoQuick precipitation and identified by NTA, Western blotting and TEM. As expected, the mean diameter of exosomes was about 120 nm (Fig. 6A). The exosomal markers (TSG101 and CD63) were also detected (Fig. 6B) and the exosomes were clearly visible by TEM (Fig. 6C). The exosomal SERPINH1, AGT and MMP7 were thereafter measured by ELISA. The exosomes level of AGT and MMP7, except SERPINH1, correlated positively with the serum level (Fig. 6D).

### The three proteins promote migration of GC cells through exosomes

**Fig. 7:**
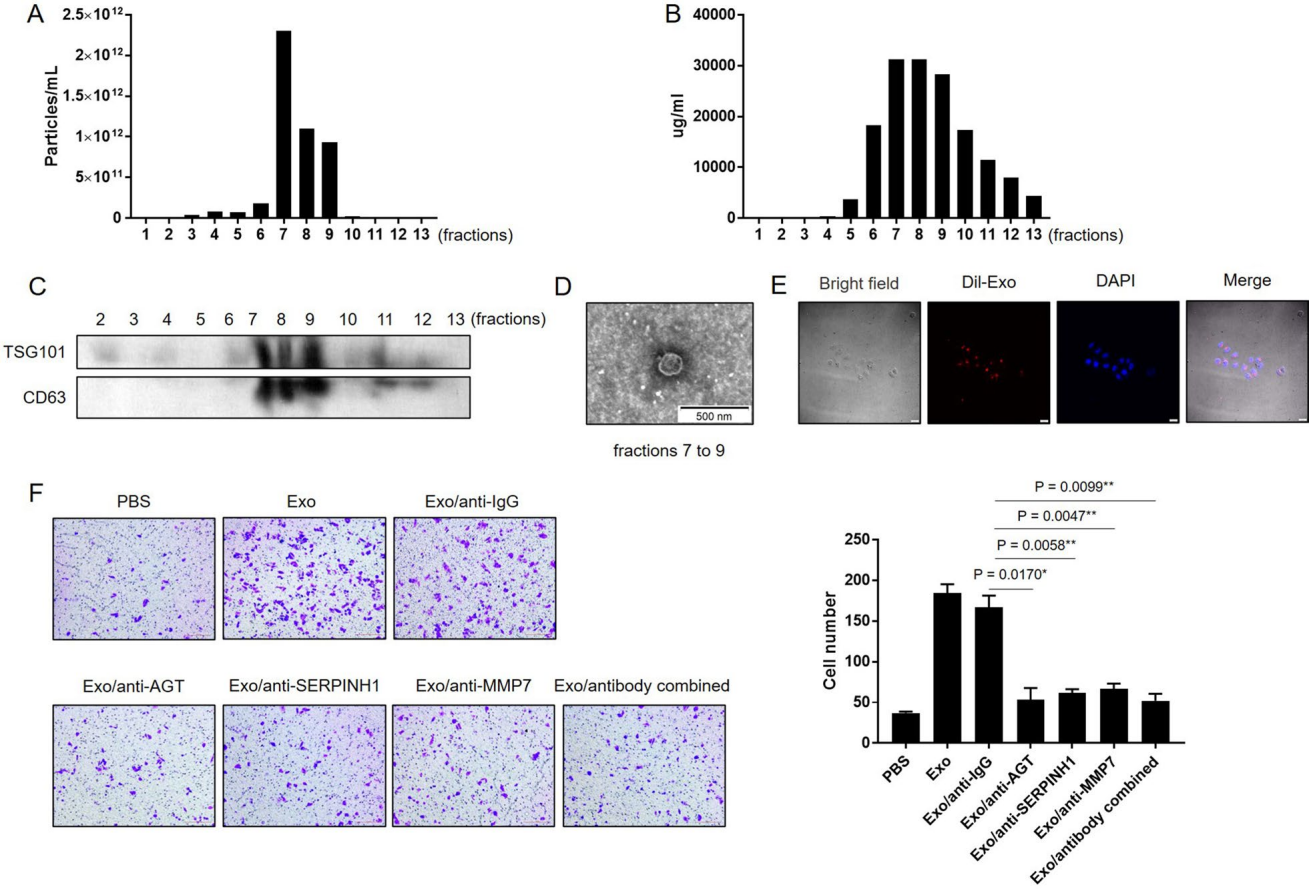
The expression level of three proteins in exosomes derived from NC and GC patients. **A**–**D** The concentration and purity of exosomes which isolated by SEC were determined by NTA, BCA, Western blotting and TEM. **E** The uptake of DiI-labeled exosomes derived from serum of GC patients by SGC7901. Nuclei were counterstained with DAPI. **F** The migration of SGC7901 treated with neutralized exosomes by antibodies. *P < 0.05, **P < 0.01

To further study the role of the three proteins in exosomes, more purer exosomes were isolated by SEC method. The concentration of particles in the SEC fractions were detected by NTA. It showed that the highest concentration of particles emerged in fractions 7–9 (Fig. 7A). In addition, the data of bicinchoninic acid assay also found the highest protein content appeared in fractions 7–9 (Fig. 7B), where the presence of exosomes were further confirmed by the exosomal markers TSG101 and CD63 (Fig. 7C). Therefore, the fractions 7–9 were collected for TEM detection and for subsequent study. The TEM scan showed that the purified exosomes were enriched in fractions 7–9 (Fig. 7D). Since the three proteins are associated with tumor invasion and TNM stage, the effect of three proteins on the migration of GC cells were studied. The exosomes were pretreated with or without neutralizing antibodies to the three proteins, and co-cultured with SGC‐7901 cells. Then the migration of GC cells was observed. Immunofluorescence staining assay indicated that exosomes can transfer into SGC‐7901 cells (Fig. 7E). The exosomes derived from serum of GC patients can promote the migration of SGC‐7901 cells. After neutralized the expression level of three proteins in exosomes with antibodies, the migration of SGC‐7901 cells was obviously suppressed (*P* < 0.05) (Fig. 7F). These results indicated that the three proteins probably promote progress of GC through exosomes.

## Discussion

Although the gold standard of diagnosis of GC is endoscopy and biopsy, the invasiveness of this method leads to poor compliance and cause a great economic burden. Blood markers play an important role in screening and aiding in diagnosis of GC due to its economic, convenient and minimally invasive characteristics. The traditional molecular biomarkers for GC include CEA [9] and CA 19–9 [10]. CEA was identified in 1965 and applied in the clinical diagnosis of GC in 1980 [11]. However, the circulating level of CEA is not a GC specific marker and is generally increased in a lot of cancers [12], and also be artificially affected by other factors, such as smoking [13]. CA 19–9 is also a commonly used in pancreatic cancer [14], colorectal cancer [15] and gastric cancer [16]. However, the low rates of sensitivity and specificity of the markers made it is insufficient for GC screening or diagnosis. Therefore, the discovery of alternative biomarkers is necessary, especially blood biomarkers.

The current usual strategy for screening biomarkers from omics data is based on the different expression folds between cancer patients and normal controls. However, this method easily overlooks many low abundant genes that are widely expressed in cancers but not in the healthy. In fact, the sensitivity of a biomarker is primarily decided by its universal expression in cancer. Therefore, in this study we established a biomarker-mining strategy based on the positive rate of a gene in all individuals with cancer, which balanced the coverage and difference of gene expression. To finding the candidate blood biomarkers, we focused on the extracellular molecules annotated by Cellular Components analysis of Gene Ontology (GO). Sixteen genes were screened out and confirmed in TCGA dataset. Further, ten of them showed high diagnostic efficiency in GSE54129 dataset. These results indicated that the novel strategy for screening biomarkers is reliable. Ideal biomarker has possible excellent properties for diagnosis, therapeutic and prognostic evaluation. We construct a prognostic risk models, which contains three genes *AGT*, *SERPINH1* and *MMP7*, based on the OS of GC patients by multivariate Cox proportional hazards regression. The three candidate biomarkers in the prognostic risk model presented a good performance in survival prediction in the training and validating datasets, and the prognostic risk model is independently associated with OS of GC patients.

To compare the efficiency of the novel method and traditional method in screening of biomarkers, the differential gene expression analysis was performed. Based on the comparability with the ‘positive ratio’-based approach and the traditional differential expression analysis, the genes that met the cutoff criteria of a fold change > 2 and an adjusted P-value < 0.05 were considered differentially expressed genes (DEGs). GSE66229, GSE27342 and GSE63089, respectively, yielded 1070, 211 and 1261 genes that were met the criteria, and 101 overlapping genes were identified. The 101 genes were annotated by Cellular Components analysis of Gene Ontology (GO), and 17 proteins were located in extracellular region: *COL12A1, COL5A2, CXCL5, AGT, LAMC2, GPRC5A, PLBD1, BGN, WNT5A, WISP1, PLAU, CXCL1, CXCL16, CHI3L1, ADAMTS12, CCL18* and *SERPINB5*. The diagnostic efficiency of these genes was assessed by ROC curve in the GSE54129 dataset. Then, there are 11 candidate genes with AUC value greater than 0.6: *COL12A1, COL5A2, CXCL5, AGT, BGN, WISP1, PLAU, CXCL1, CHI3L1, ADAMTS12* and *CCL18*. The 11 genes were used for constructing of cox regression model in training dataset GSE15459. Among the 11 genes, *AGT* and *BGN* displayed significant prognostic values. Risk score =  (0.184 × expression value of *AGT*) +  (0.487 × expression value of *BGN*). Further, when evaluating the prognostic power of this model in validating dataset TCGA, patients in the high-risk group suffered the tendency of poorer OS than those in the low-risk group, however, there was on statistical difference (*P* = 0.063). These results indicated that the novel strategy may screen out more biomarkers with high efficiency.

AGT was rarely studied in GC. Previous studies focus on the association between AGT gene polymorphism and Helicobacter pylori infection-related GC or high-salt diet-related GC [17, 18]. Recent study reports that AGT was aberrantly methylated and associated with prognosis in GC [19]. Our study revealed that AGT was high expressed in serum and exosomes derived from GC patients. It with sensitivity 43.15% and specificity 82.35% for distinguishing GC patients from controls. High-AGT patients showed larger tumor size, more depth of tumor invasion and more advanced TNM stage. Further, the serum level of AGT was significantly decreased one month after radical operation. These results suggested that AGT has the potential to be a non-invasive biomarker in GC. Some studies, based on bioinformatics analysis of public database, reported that SERPINH1 was up-regulated in GC tissues [20–24]. Tian et al. revealed that SERPINH1 expression was significantly higher in the GC cell lines than in the normal gastric mucosal cell line. SERPINH1 regulates EMT and GC progression via the Wnt/β-catenin pathway [25]. SERPINH1 is considered to be the target gene of antitumor miR-148a-5p in GC cells. Knockdown of SERPINH1 resulted in inhibition of the aggressive phenotype of GC cells [26]. We focus on the concentration of SERPINH1 in serum and the results revealed that the level of serum SERPINH1 had high diagnostic efficacy in GC, with the AUC of 0.7355 (sensitivity 60.27% and specificity 79.52%). High-SERPINH1 patients showed more depth of tumor invasion and more advanced TNM stage than the low-SERPINH1 ones. The serum level of SERPINH1 was also significantly decreased one month after radical operation. These data indicated that SERPINH1 could be considered as a serum marker in diagnosis and prognosis assessment of GC. Previous studies confirmed that EGFR/MMP7 signaling pathway was activated in GC and played a role in GC metastasis [27–29]. MMP7 was directly or indirectly regulated by some microRNA and participated in GC metastasis [27, 30]. However, the content of MMP7 in serum, especially in exosomes, were not studied sufficiently. In the present study, we found that MMP7 showed high sensitivity and specificity in distinguishing GC patients from controls, and high expression of MMP7 was related to the malignant characteristics of GC. When evaluating the prognosis of GC patients by combination of the three proteins in serum, it still showed good performance. These results indicated that the three candidate biomarkers showed excellent potential as the GC blood markers.

In recent years, extracellular exosomes have emerged as a novel biomarker pool for discovering the diagnostic and prognostic signatures of many diseases, especially in regard to cancer diagnosis [31, 32]. Proteins which carried by exosomes are ideal blood biomarker candidates, since they bear the traits of the original tissue and the signatures of the homing organs or cells [33, 34]. Unsurprisingly, several exosomal proteins have been demonstrated as potential diagnostic markers in GC. Yen et al. analyzed TGF-beta1 expressions in the exosomes isolated from the gastroepiploic veins of 61 GC patients by ELISA and revealed that increased exosomal TGF-β1 expression level was correlated with lymph node metastasis [35]. In the present study, the exosomes level of AGT and MMP7 were correlated positively with the serum level. Although there is a trend of positive correlation between exosomes level and serum level of SERPINH1, however, there was no statistical significance. This may be due to the small sample size. In addition, antibodies of the three proteins inhibited migration of GC cells, which mediated by exosomes derived from GC patients. Exosomal contents are relative stability and the proteins in blood derived exosomes are suitable as diagnostic and prognostic biomarkers. The results revealed that exosomal AGT, SERPINH1 and MMP7 may serve as biomarkers for gastric cancer diagnosis and prognosis and involved in GC progression.

## Conclusions

In summary, using the novel biomarker-mining strategy, we identified three genes (*AGT*, *SERPINH1* and *MMP7*) expression profiles with good performance in diagnosis and prognosis of GC patients. We confirmed the diagnostic and prognostic value of the three-gene signature not only in public datasets but also in clinical serum samples. In addition, we detected the expression level of the three candidate biomarkers in exosomes derived from serum, and found that these proteins promote migration of GC cells through exosomes. These findings suggested that AGT, SERPINH1 and MMP7 have great potential as diagnostic and prognostic blood biomarkers and involved in GC progression.

## Data Availability

The data sets analyzed in the present study are publicly available data from The Cancer Genome Atlas (TCGA) and Gene Expression Omnibus (GEO). The original contributions presented in the study are included in the article. Further inquiries can be directed to the corresponding authors.
